# Significance of Some Non-Invasive Biomarkers in the Early Diagnosis and Staging of Egyptian Breast Cancer Patients

**DOI:** 10.31557/APJCP.2020.21.11.3279

**Published:** 2020-11

**Authors:** Tarek MK Motawi, Nadia I Zakhary, Hebatallah A Darwish, Hassan Abdullah, Samer A Tadros

**Affiliations:** 1 *Department of Biochemistry, Faculty of Pharmacy, Cairo University, Cairo, Egypt. *; 2 *Department of Cancer Biology, National Cancer Institute, Cairo University, Cairo, Egypt. *; 3 *Pharmacology, Toxicology and Biochemistry Department, Faculty of Pharmaceutical Sciences and Pharmaceutical Industries, Future University in Egypt (FUE), Cairo, Egypt. *; 4 *Department of Surgical Oncology, National Cancer Institute, Cairo University, Cairo, Egypt. *; 5 *Department of Biochemistry, Faculty of Pharmacy, October University for Modern Sciences and Arts (MSA), October, Egypt. *

**Keywords:** Breast cancer, early diagnosis, CEACAM1, visfatin and resistin

## Abstract

**Introduction:**

Breast cancer is one of the most relevant malignancies among women. Early diagnosis and accurate staging of breast cancer is important for the selection of an appropriate therapeutic strategy and achieving a better outcome.

**Aim:**

This study aimed to explore the significance of some non-invasive biomarkers in the early diagnosis and staging of Egyptian breast cancer patients.

**Subjects and Methods:**

A total of 135 female patients with physically and pathologically confirmed breast cancer and 40 unrelated controls as well as 40 patients with benign breast mass were enrolled in this study. The malignant breast cancer group was further divided into four groups according to tumor size. Serum levels of carcinoembryonic antigen-related cell adhesion molecule-1 (CEACAM1), resistin and visfatin were determined by enzyme immunoassay.

**Results:**

Elevated levels of CEACAM1, resistin and visfatin were observed in breast cancer patients when compared with normal control and benign groups. The cutoff values, sensitivities and specificities of these biomarkers were appropriate for the discrimination of breast cancer from controls. Additionally, the serum levels of visfatin increased positively with tumor size and consequently with breast cancer stages.

**Conclusion:**

CEACAM1, resistin and visfatin are valuable in early diagnosis of breast cancer, with visfatin being preferentially used in staging.

## Introduction

Breast cancer represents the most common type of malignancy among women and one of the important contributors to the global health burden. It has been reported that additional cases are occurring in less developed (883,000 cases) than more developed regions (794,000) and that the rate is rapidly rising in less developed (Ito and Matsuo, 2016). In Egypt, Breast cancer occupies 33.8% in upper Egypt, 26.8% in middle Egypt and 38.7% in lower Egypt from all cancer cases (Ibrahim et al., 2014). It represents 35.1% of new cancer cases of females in Egypt. Moreover, it’s the second most common cancer and it’s ranked the second cancer type in mortality (Bray et al., 2018).

Stage at which diagnosis occurs is an important predictor of breast cancer survival and quality of life, so there is an urgent need for early diagnostic indicators (Gao et al., 2013).

CEACAM1, is a transmembrane glycoprotein synthesized in endothelial cells, B cells, interleukin activated T cells, and epithelial cells of the gastrointestinal tract. It is also synthesized in the breast, prostate and endometrium (Kim et al., 2019). CEACAM1 was found to regulate tumor development by affecting cellular proliferation, apoptosis, angiogenesis, invasion and migration (Beauchemin and Arabzadeh, 2013). Changes of CEACAM1 expression in different types of malignancies were contradictory; some reports pointed to a partial reduction or loss of CEACAM1 expression in breast cancer (Wang et al., 2011), while others demonstrated that CEACAM1 was expressed to the same extent in both normal and malignant breast tissues (Takeuchi et al., 2019). Nevertheless, Yang et al. have showed that there was an elevation of CEACAM1 levels in breast cancer patients, clarifying its role in the diagnosis of breast cancer (Yang et al., 2015b). Since the aberrant CEACAM1 expression may play a pivotal role in tumorigenesis (Beauchemin and Arabzadeh, 2013), it is therefore of interest to investigate CEACAM1 expression in breast cancer patients for more details.

Adipokines, as resistin and visfatin are produced by different fat depots, including sub-cutaneous, visceral and mammary adipose tissues and may act on breast tissue in an endocrine, paracrine and autocrine manner (Kratzsch et al., 2018). 

Resistin is also known as adipocyte-secrete factor or found in inflammatory zone 3 (FIZZ3) (Acquarone et al., 2019). It is involved in autocrine and paracrine cell signaling and may provide novel insights into the mechanisms of cancer initiation, progression, regression and persistence (Dalamaga, 2014). Resistin can directly act on cancer cells by stimulating specific signaling pathways that are important components of the cancer-promoting machinery such as TLR4 receptor stimulation. Furthermore, it can enhance cancer progression by inducing cell proliferation mediated by the activation of the PI3K and MAPK pathways (Park et al., 2014). Resistin may also act indirectly through the activation of transcription factors such as NF-κB that leads to the activation of proinflammatory genes involved in the initiation, promotion, and progression of carcinogenesis (Howe et al., 2013). 

Visfatin, also known as pre-B cell colony-enhancing factor (PBEF) and nicotinamide phosphoribosyl-transferase (NAMPT), plays an important role in the inflammation and in a variety of metabolic and stress responses as well as in the cellular energy metabolism (Hu et al., 2013). Although, studies have suggested that extracellular visfatin influences signaling pathways (Park et al., 2014), no specific receptor has yet been identified for visfatin and the mechanism of its contribution to cancer development and progression is still under debate (Garten et al., 2015). Whyte et al. pointed out the role of visfatin in upregulating AKT/PI3K and ERK/MAPK signaling pathways which are key components of cell proliferation and survival (Carbone et al., 2017). Park et al. found that visfatin increased the levels of NF-κB p65 as well as Notch1, suggesting a role for NF-κB p65 as a positive regulator of Notch1 induction by visfatin in breast cancer cells (Park et al., 2014). Likewise, Gholinejad et al. have implicated that increased visfatin levels may augment breast cancer development and attenuate treatment efficiency in breast cancer patients (Gholinejad et al., 2017).

In view of that, this study was designed to evaluate the serum levels of CEACAM1, resistin, visfatin in order to explore their significance in the early diagnosis and staging of Egyptian breast cancer patients. It also aimed at investigating the possible correlations between these biomarkers and determining their usefulness as diagnostic non-invasive tool in breast cancer.

## Materials and Methods

*Subjects and methods*

Study participants: This study comprised 135 Egyptian females, with ages ranged from 18 to 72 years, suffering from malignant breast cancer and selected from the early detection unit at National Cancer Institute (NCI), Cairo University, Egypt. They were recruited between April 2015 to January 2017. Patients did not receive radiotherapy or chemotherapy before surgery and during sampling. The study also included an age matched control group comprising 40 apparently healthy females, with no history of malignancy. Additionally, the benign group included 40 patients diagnosed with benign breast mass. Physical examinations and diagnosis were processed by physicians in the Medical Oncology Unit of the NCI, where routine clinical and pathological examinations were done. Once the diagnosis is confirmed, clinical staging of the disease was done after mastectomy according to the TNM classification of the American Joint Committee on Cancer (AJCC) TNM system. The malignant breast cancer group was further divided into three groups according to the tumor size: T1 (less than 20 mm), T2 (20 – 50 mm), T3 and T4 (greater than 50 mm), where each group included 45 breast cancer patients. Informed consent was obtained from the participating patients in adherence with the guidelines of the ethical committee of the National cancer institute (NCI), Cairo, Egypt. The study was also approved by the ethical committee of faculty of Pharmacy, Cairo University, Egypt, and in accordance with the Declaration of Helsinki principles (Code: Bio 7.4.1).

Sample collection: Whole blood was collected from recruited subjects by trained laboratory technicians in vacutainers and the serum was obtained by centrifugation of vacutainers at 5000 rpm for 15 minutes at 25°C. The separated serum was stored at -80^o^C until used for further analysis. 

Determination of serum CEACAM1, resistin and visfatin levels: the serum levels were measured using ELISA technique (Bioassay Technology Laboratory, Korain Biotech CO.LTD., Shanghai, China) following the manufacturer’s instructions.

*Statistical analysis*

The statistical data were reported as mean ± SD, frequencies (number), and percentages when applicable. Comparison of numerical variables between the studied groups were performed using student’s t test to compare independent samples from two groups when the samples were normally distributed and the Mann-Whitney U test to compare independent samples when the samples are not normally distributed. The Receiver Operation Characteristic (ROC) curve was plotted to determine the cutoff values and to analyze the diagnostic utility of CEACAM1, resistin and visfatin. Cutoff values were selected at a balance between highest possible sensitivity and specificity. The P values less than 0.05 were considered statistically significant. Statistical analysis and comparison between groups were performed using Graphpad prism version 6.01 (Graphpad software, CA, USA) for Microsoft Windows.

## Results

*Characteristics of the study participants*

Table 1 showed the count, age and tumor size of studied participants. 

*Serum level of CEACAM1 in different investigated groups*

Results of Table 2 showed significant increase in serum level of CEACAM1 at different stages of breast cancer patients compared with the benign and control groups. It is worthy to note that the level of CEACAM1 was significantly higher in T1 relative to T2 group. Additionally, T3 and T4 group showed significantly higher serum values than those of T2 group. 

*Serum level of resistin in different investigated groups*

Results in Table 3 showed that the serum level of resistin in different stages of breast cancer patients was significantly higher when compared with the benign and control groups. Moreover, it was significantly higher in T1 group when compared with T2 group. Additionally, there were significant increments in the levels of T3 and T4 groups relative to T2 group.

*Serum level of visfatin in different investigated groups*

Results in Table 4 showed that the serum levels of visfatin in different stages of breast cancer patients were significantly higher when compared with the benign and control groups. Moreover, there was a gradual increase in serum levels of visfatin relative to tumor size (T1<T2<T3 and T4 groups).

*Receiver-operating characteristic (ROC) curve analysis of serum CEACAM1*

**Figure 1:**
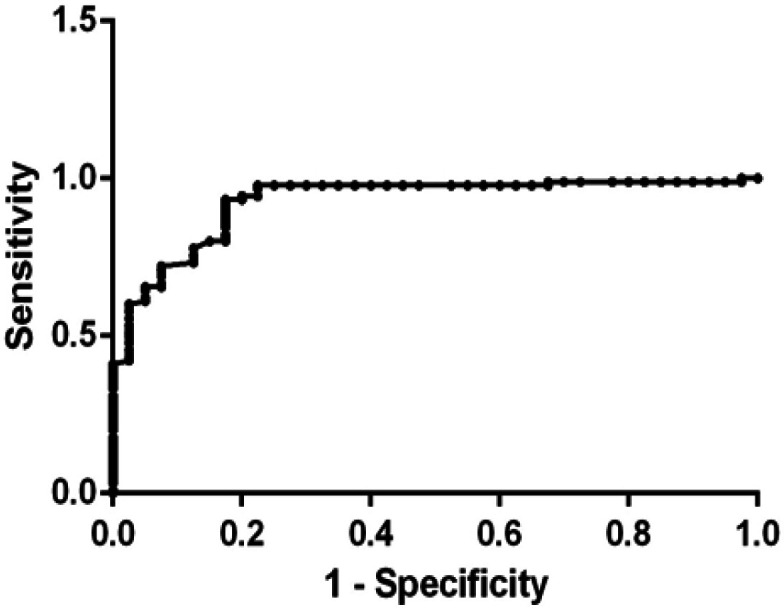
Receiver Operating Characteristic (ROC) Curve of Serum CEACAM1

According to the ROC curve (figure 1), serum CEACAM1 cutoff value ≥ 2.22 ng/ml can be used as a cutoff point at which 93.3% of breast cancer patients (T1 and T2 stages) can be early diagnosed correctly, but 17.5% of normal subjects are false positive. Thus, the sensitivity was 93.3% while the specificity was 82.5% at P ≤ 0.001 with area under the curve equal to 0.92.

*Receiver-operating characteristic (ROC) curve analysis of serum resistin*

**Figure 2:**
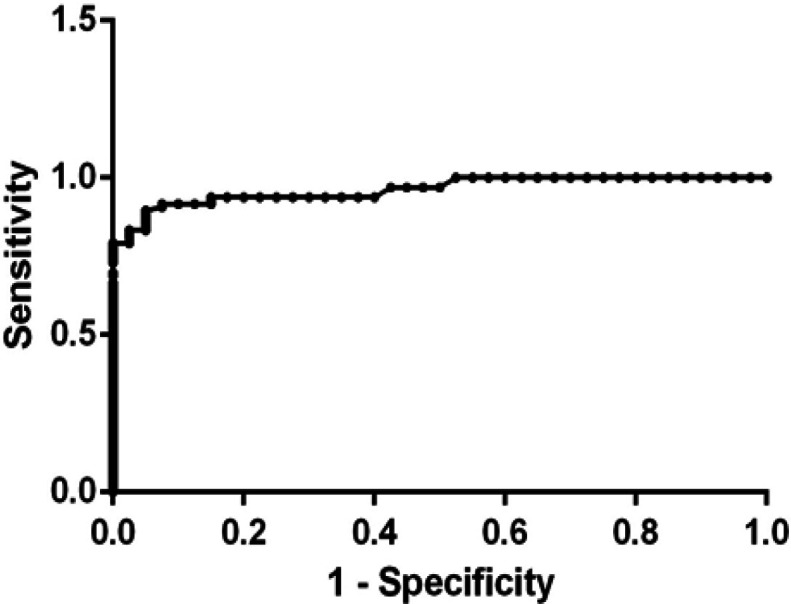
Receiver Operating Characteristic (ROC) Curve of Serum Resistin

Based on the ROC curve (Figure 2), serum resistin cutoff value ≥ 737.6 ng/L can be used as a cutoff point at which 83.3% of breast cancer patients (T1 and T2 stages) can be early diagnosed correctly, but 2.5% of normal subjects are false positive. The sensitivity was thus 83.3% while the specificity was 97.5% at P ≤ 0.001 with area under the curve equal to 0.96.

*Receiver-operating characteristic (ROC) curve analysis of serum visfatin*

**Figure 3:**
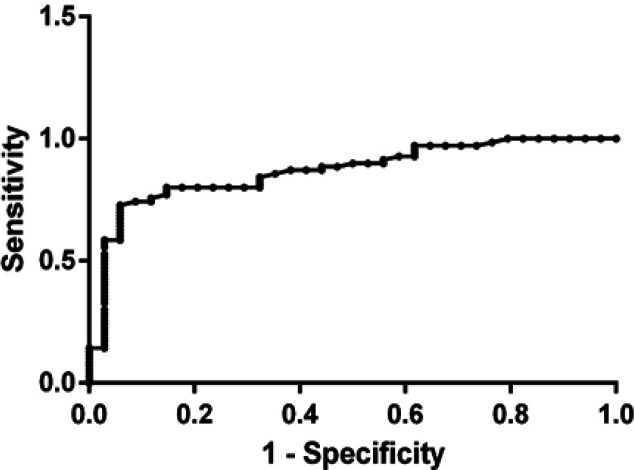
Receiver Operating Characteristic (ROC) Curve of Serum Visfatin

As shown in Figure 3, serum visfatin cutoff value ≥ 9.06 ng/ml can be used as a cutoff point at which 80% of breast cancer patients (T1 and T2 stages) can be early diagnosed correctly but 14.71% of normal subjects are false positive. Accordingly, the sensitivity was 80% while the specificity was 85.2% at P ≤ 0.001 with area under the curve equal to 0.86.

*Correlations of CEACAM1, resistin and visfatin with other studied parameters in breast cancer patients*

It was obvious from Table 5 that there was a significant positive correlation between resistin and CEACAM1. In addition, there were positive correlations between visfatin and tumor size in breast cancer patients.

**Table 1:** Age and Histopathological Features of Studied Groups

Group Subgroup	Controls	Benign tumors	Malignant breast cancer		
			T1	T2	T3 and T4
Count (Females)	40	40	45	45	45
Age (Years)	36.85 ± 13.09	40.35 ± 13.80	39.02 ± 14.04	37.4 ± 12.76	41.13 ± 14.04
Tumor size (mm)	0	15.85 ± 6.26	14.29 ± 2.87	36.37 ± 9.00	69.69 ± 9.94

Data were expressed as mean ± SD

**Table 2:** Serum CEACAM1 Concentration in Control, Benign, T1, T2 and T3 and T4 Groups

Group	Stage	(N)	CEACAM1 concentration (ng/ml)
Controls		40	1.72 ± 0.45
Benign tumors		40	1.92 ± 0.53
Malignant breast cancer	T1	45	3.81 ± 1.01 ^a^
	T2	45	2.76 ± 0.47 ^ab^
	T3 and T4	45	3.90 ± 0.99 ^ac^

Data are presented as mean ± SD; N, number of cases; ^a^, Significantly different from control and benign groups; ^b^, Significantly different from T1 group; ^c^, Significantly different from T2 group.

**Table 3:** Serum Resistin Concentration in Control, Benign, T1, T2 and T3 and T4 Groups

Group	Stage	(N)	Resistin concentration (ng/L)
Controls		40	434.3 ± 105.9
Benign tumors		40	532.1 ± 106.2
Malignant breast cancer	T1	45	1031.5 ± 179.6 ^a^
	T2	45	823.7 ± 98.3 ^ab^
	T3 and T4	45	1008.1 ± 210.0 ^ac^

Data are presented as mean ± SD; N, number of cases; ^a^, Significantly different from control and benign groups; ^b^, Significantly different from T1 group; ^c^, Significantly different from T2 group.

**Table 4:** Serum Visfatin Concentration in Control, benign, T1, T2 and T3 and T4 Groups

Group	Stage	(N)	Visfatin concentration (ng/ml)
Controls		40	7.33 ± 1.14
Benign tumors		40	8.38 ± 2.92
Malignant breast cancer	T1	45	12.04 ± 3.01 ^a^
	T2	45	15.48 ± 3.45 ^ab^
	T3 and T4	45	18.68 ± 3.92 ^abc^

Data are presented as mean ± SD; N, number of cases; ^a^, Significantly different from control and benign groups; ^b^, Significantly different from T1 group; ^c^, Significantly different from T2 group.

**Table 5:** Correlations of CEACAM1, Resistin and Visfatin, in Breast Cancer Patients with Other Studied Parameters

Parameter		Resistin	Visfatin	CEACAM1
Age	r	0.034	0.144	0.116
	p	NS	NS	NS
Tumor Size	r	-0.056	0.622	0.025
	p	NS	<0.05	NS
Resistin	r	-	0.026	0.498
	p	-	NS	<0.01
Visfatin	r	0.026	-	0.071
	p	NS	-	NS
CEACAM1	r	0.498	0.071	-
	p	<0.01	NS	-

r, Spearman's rank correlation coefficient

## Discussion

Breast cancer is the most common neoplasm in women and has the highest associated mortality rate. Rapid detection programs can provide relatively early diagnosis and increase the chances of survival (Bayo et al., 2018). The present study aimed to determine the usefulness of some non-invasive serum biomarkers in the early diagnosis and staging of Egyptian breast cancer patients.

CEACAM1 has been shown to play an essential role in lumen formation during morphogenesis and even could revert breast cancer cells to a normal phenotype (Kirshner et al., 2003). Herein, there were elevated levels of serum CEACAM1 in all stages of breast cancer compared with normal controls and benign breast lesions. These results agreed with Yang et al., (2015b) who reported increased serum CEACAM1 concentrations in malignant breast cancer group with a predictive cutoff value compared to healthy controls. On the contrary, a study on breast cancer cell line demonstrated a systematic down-regulation of CEACAM1 and suggested that restoration of CEACAM1 expression may be a promising strategy for the treatment of breast cancer (Yang et al., 2015a). Similarly, Wang et al. reported that 65% of the studied cancer tissues had lower CEACAM1 expression than that of adjacent tissues (Wang et al., 2011). It is possible that the different expression levels of CEACAM1 are due to different populations and different environments. Another possible explanation is the diversity of the expressed isoforms of CEACAM1 (Yang et al., 2017).

Resistin is an inflammatory marker that was initially described as an adipocyte-derived cytokine and has been studied extensively for its role in inflammation and cancer (Gong et al., 2016). As shown in the present study, serum level of resistin in different stages of breast cancer patients was significantly higher when compared with the benign and control groups. In tune, Li et al., (2017) found elevated serum resistin levels in breast cancer patients compared with controls and recommended the investigation of its specificity for improving the diagnosis and prognosis of breast cancer. Another study also showed that serum resistin was elevated in breast cancer compared with healthy controls and benign breast lesions pointing to its importance as a diagnostic biomarker (Assiri and Kamel, 2016). Furthermore, increased levels of resistin were reported in breast cancer Caucasian patients, and these levels were correlated with breast tumor growth and aggressiveness (Vallega et al., 2016). An earlier study stated that the mean serum resistin was significantly higher in breast cancer cases than in controls and patients with benign breast lesions (Dalamaga et al., 2013). Meanwhile, other findings indicated that resistin levels were inversely related to breast cancer risk in premenopausal women, supporting a protective role of resistin for these patients (Georgiou et al., 2016).

It is worthy to note that this study showed a positive correlation between resistin and CEACAM1 in breast cancer patients. This finding could be explained on the basis that the elevated expression of CEACAM1 in neutrophils is involved in mediating the inflammatory response by producing chemokines and cytokines such as resistin (Najjar and Russo, 2014).

Visfatin is a novel adipokine and proinflammatory cytokine which is implicated in breast cancer progression (Gholinejad et al., 2017). Herein, the serum level of visfatin in different stages of breast cancer patients was significantly higher when compared with the benign and control groups. Moreover, there was a gradual increase in visfatin concentration relative to tumor size. In harmony, elevated visfatin levels in serum and tumor tissue of breast cancer patients have been reported by Zhu et al., (2016) and were associated with poor patient survival. Assiri and Kamel (2016) also showed that serum levels of visfatin were significantly higher in breast cancer compared to normal control and benign groups. Likewise, its level was also significantly higher in advanced TNM stage, tumor size, LN invasion, histological grade and negative Estrogen Receptors (ER) or Progesterone Receptors (PE) cases. Another study showed that serum visfatin was elevated in postmenopausal breast cancer patients compared with controls. Similarly, an earlier study found that the mean serum visfatin level was significantly higher in cases than in control participants and could therefore be used as a biomarker for postmenopausal breast cancer (Christodoulatos et al., 2019).

Eventually, the current study explored the potential utility of CEACAM1, resistin and visfatin as early non-invasive biomarkers. Their calculated cutoff values, sensitivities and specificities were appropriate for the discrimination of breast cancer from controls. Moreover, visfatin levels were relatively correlated with the progression of breast cancer and could be a helpful tool in staging and follow-up of the progress of the disease in different breast cancer stages.
